# An update on current EPAs in graduate medical education: A scoping review

**DOI:** 10.1080/10872981.2021.1981198

**Published:** 2021-09-26

**Authors:** Lu Liu, Zhehan Jiang, Xin Qi, A’Na Xie, Hongbin Wu, Huaqin Cheng, Weimin Wang, Haichao Li

**Affiliations:** a Institute of Medical Education, Peking University, Beijing, China; b National Center for Health Professions Education Development, Peking University, Beijing, China; c Peking University First Hospital, Beijing, China

**Keywords:** Entrustable professional activities, postgraduate medical education, assessment, scoping review

## Abstract

The purpose of this scoping review is to update the recent progress of EPAs research in GME, focusing on the topical concern of EPAs effectiveness, and to provide a reference for medical researchers in countries/regions interested in introducing EPAs. Guided by Arksey and O’Malley’s framework regarding scoping reviews, the researchers, in January 2021, conducted a search in five databases to ensure the comprehensiveness of the literature. After the predetermined process, 29 articles in total were included in this study. The most common areas for the implementation and evaluation of EPAs were Surgery (n = 7,24.1%), Pediatric (n = 5,17.2%) and Internal medicine (n = 4,13.8%), a result that shows a relatively large change in the research trend of EPAs in the last two years. Prior to 2018, EPAs research focused on internal medicine, psychiatry, family medicine, and primary care. The articles in the category of EPAs implementation and evaluation had four main themes: (1) validation of EPAs (n = 16,55.2%); (2) describing the experience of implementing EPAs (n = 11,37.9%); (3) examining the factors and barriers that influence the implementation and evaluation of EPAs (n = 6,20.6%); and (4) researching the experiences of faculty, interns, and other relevant personnel in using EPAs. Training programs were the most common EPAs implementation setting (n = 26,89.6%); direct observation and evaluation (n = 12,41.4%), and evaluation by scoring reports (n = 5,17.2%) were the two most common means of assessing physicians’ EPA levels; 19 papers (65.5%) used faculty evaluation, and nine of these papers also used self-assessment (31.0%); the most frequently used tools in the evaluation of EPAs were mainly researcher-made instruments (n = 37.9%), assessment form (n = 7,24.1%), and mobile application (n = 6,20.7%). Although EPAs occupy an increasingly important place in international medical education, this study concludes that the implementation and diffusion of EPAs on a larger scale is still difficult.

## Introduction

Entrustable Professional Activities (EPAs), proposed by ten Cate in 2005 [1], are observable, measurable, and work-based professional practices that can be entrusted to a sufficiently competent learner or professional [2]. As a valuable way to conduct competency-based medical education, EPAs offer a beneficial yet robust framework for constructing assessments based on direct observations or reviews of performance along with formative feedback [3]. Nowadays, EPA is regarded as a valid assessment tool in Graduate Medical Education(GME) in countries such as the USA, Canada, and Australia. The Canadian Medical Education Directives for Specialists (CanMEDS) framework defines seven core-competency areas in GME that can be mapped to EPAs [4]. In 2014, the Association of American Medical Colleges (AAMC) published a list of 13 core EPAs that all medical students should be able to perform before starting residency[5]. Further, from January 2017, EPA assessments have replaced mid- and end- semester physician assessments for general practice physicians in South Australia [6].

The American Board of Pediatrics(ABP) identified 17 EPAs based on competencies and milestones in 2018, and defined them as fundamental pediatrician abilities [7]. In the same year, a pilot 2-year project was launched by the American Board of Surgery(ABS) using 5 EPAs to evaluate physicians’ capacity in general surgery residency programs [8]. As a result, there has been a perceptible shift of EPAs research from introducing the development principles and experience, to validating and assessing the efficacy of the framework in clinical settings.

The idea of the present study was inspired by the review article by O’Dowd et al., [9] where the authors selected 49 studies published during 2011–2018 and outlined imperative steps for the EPA improvement: (i) devising best practice guidelines for the EPAs development, (ii) shedding lights on the methodological quality of research on the EPAs literature, and (iii) examining the implementation of EPAs in the curriculum. While the research is valuable, it focuses mainly on the development of EPAs based on few clinical cases, thus providing limited information on current implementation and assessment of the EPAs in GME. Thus, we conducted this review to provide an up-to-date overview of the literature on EPA implementation and assessment in GME. We believe a scoping review is necessary as it expands broader research area, rather than simply assessing the quality of extant research/practice like what O’Dowd et al provided[10]. As the topic per se is relevant to GME in the worldwide, a report conducted to guide the practice globally is helpful. Since more EPAs-related papers have been published in the last two years (i.e., 2019 to 2020), compared with the previous 7-year window, it’s needed to update the recent progress. As the EPAs’ effectiveness is the primary focus in the present context, we emphasize on the implementation and assessment of EPAs and assert that the time is now to undertake such an analysis.

## Methods

### Research question

This study aims to clarify four questions: (1) general characteristics of eligible studies; (2) common ways to implement and assess EPAs in GME; (3) results of EPAs practice in GME; (4) challenges of EPAs practice in GME.

Guided by the framework presented by Arksey and O’Malley[11], we conducted a scoping review of the implementation and assessment of EPAs in GME, to synthesize and disseminate research findings, identify the areas where more research is needed, then provide guidance for parties involved in implementing and evaluating EPAs A scoping review is defined as a type of research synthesis that aims to ‘map the literature on a particular topic or research area and provide types and sources of evidence to inform practice, policy making, and research’ [12]. This review consists of five key phases: (1) identifying the research question; (2) identifying relevant studies; (3) filtering the studies to match the research inquiry; (4) organizing/charting the data; and (5) collating, analyzing, and reporting the results.

### Identification of relevant studies

In January 2021, the research team searched literature from 2019 to 2020 in five electronic databases, including Medline, CINAHL, Scopus, PsycINFO and Web of Science with the keywords ‘Entrustable professional activity/activities or EPA’, and varied them by the entry formats (see Appendix). To cover those only seen in the ‘gray’ literature, we also searched the key terms in Google and Google Scholar, screened the top 200 results.

### Selecting the studies to be included

The topic-EPA-is the core criterion for selecting studies in this study. Two screening levels took place: a title and abstract review for articles that met pre-defined criteria was conducted, followed by a full-text review of articles that fit those criteria. The eligibility criteria used to screen abstracts and full texts are listed in Table 1.

**Table 1.** Study eligibility criteria

Inclusion criteria	The following articles were included: ·Written in English ·Published during 2019–2020 ·Focuses on the implementation and/or assessment of EPAs ·Target on postgraduate physicians
Exclusion criteria	The following articles were excluded: ·Non‐English articles ·Focus of the article is not on the EPAs per se (e.g., milestones, capabilities) ·Subject is physician in other healthcare professionals (i.e., a nurse) or a physician not in the postgraduate stage ·Absence of original data regarding the implementation or assessment of EPAs

**Figure 1.**
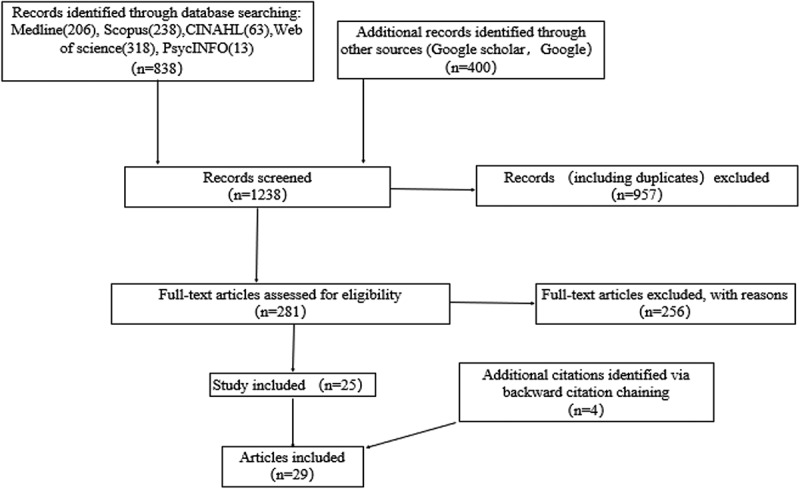
Scoping review process

Three members conducted the selection process, two authors (LL, ZHJ) worked together to screen the articles by examining the titles and abstracts. If the information was inadequate to make a judgment, the full text would be reviewed prior to the inclusion/exclusion decision. To supplement the search, members also scanned the references of included studies. Controversial papers (i.e., there was a conflict during our judgment) were discussed in further meetings with the third author (XQ). The screening process and results are shown in Figure 1.

### Data extraction

Data were gathered in a single spreadsheet and then imported into Microsoft Excel 2016 (Microsoft Corporation, Redmond, WA) for intensive reading. Information was extracted on the year of publication, country of study, medical specialty, method of implementation and assessment, grade of the physicians, results of EPAs practice, challenges of EPAs practice. Like the inclusion process, the two researchers (LL, ZHJ) completed the data extraction and collection, and the controversies were discussed in meetings until a consensus was reached.

### Collating, summarizing and reporting results

Data analysis involved both quantitative and qualitative synthesis by a team consisting of a combination of statisticians, physicians, medical educators with doctorates. The first author (LL) thematically analyzed the data and extracted data to answer the research question and meet the objectives of this scoping review. ZHJ and LL discussed findings, which were then discussed with the rest of the research team (HCL, HQC and ANX). Descriptive statistics are provided to summarize the data. Our syntheses of researchers’ perspectives were by no means explicitly being described by researchers; they represented our interpretations and reanalysis of existing research, instead.

## Results

### General characteristics of eligible studies

We included 29 studies published in 2019(n = 13,45%) and 2020(n = 16,55%). As can be seen in Table 2, geographically, first authors were from the USA(n = 19,65.5%), Canada(n = 3,10.3%), India(n = 2,6.8%), Netherlands(n = 2,6.8%), Australia(n = 1,3.4%), Ireland(n = 1,3.4%) and Germany(n = 1,3.4%). Articles were published in 21 journals, including *Journal of Surgical Education* (n = 6, 20.6%), *Journal of Graduate Medical Education*(n = 3,10.3%) and *Academic Pediatrics* (n = 2, 6.8%) publishing the most. Studies reported the implementation and assessment of EPAs across thirteen different specialties, the most common were Surgery(n = 7,24.1%), Pediatric(n = 5,17.2%) and Internal medicine(n = 4,13.8%). The grade of physicians varied across studies (see Table 2), ‘Residents’ (n = 20; 68.9%) and ‘Interns’(n = 4,13.8%) were how participants were most often described.

**Table 2.** General characteristics of eligible studies

Characteristics	No. (%)	References
**Study location**		
USA	19(65.5)	[14–17–19–21–23–24–26–30–32–34–36–39]
Canada	3(10.3)	[18,37,40]
India	2(6.8)	[22,31^]^
Netherlands	2(6.8)	[25,38^]^
Australia	1(3.4)	[^6]^
Ireland	1(3.4)	[^13]^
Germany	1(3.4)	[33]
**Specialty**		
Surgery	7(24.1)	[16–21–23–24–34–36^]^
Pediatric	5(17.2)	[14,17,25,30,39^]^
Internal medicine	3(13.8)	[15,20,28^]^
Community medicine	2(6.8)	[6,22]
Medical Oncology	2(6.8)	[37,40]
Otorhinolaryngology	1(3.4)	[31]
Radiology	1(3.4)	[38]
Dentistry	1(3.4)	[32]
Emergency medicine	1(3.4)	[18]
Physical Medicine and Rehabilitation	1(3.4)	[19]
Anesthesiology	1(3.4)	[13]
Pathology	1(3.4)	[27]
Psychiatry	1(3.4)	[26]
Multiple specialties	2(6.8)	[29,33]
**Grade of physicians**		
Residents	20(68.9)	[16–18–21–23–27–30–31–33–40^]^
Interns	4(13.8)	[14,15,28,29]
Physicians	2(6.8)	[6,13]
Fellows	1(3.4)	[17]
Not specified	2(6.8)	[22,32^]^
**Research purpose**		
Verify the reliability and validity of EPAs (including framework, tools, app, instruments, system, curriculums)	16(55.2)	[6–15–19–22–27–32–34–35–37]
Present practices of EPAs implementation	11(37.9)	[14,15,20,21,24,25,28,30,31,35,40^]^
Survey on EPAs experience of related personnel	6(20.6)	[13,25,29,33,36,39^]^
Gather information about factors and barriers affecting EPAs’ implementation	5(17.2)	[26–36–39^]^
·Percentages do not sum to 100%, as one study may report more than one purpose		

The current review demonstrates that the number of published papers addressing the implementation and assessment of EPAs, has dramatically increased within the recent two years; the changes take place in the research’s general characteristics entailed in Table 2. In the present review, surgery and pediatric are the two major areas addressed in the studies related to the implementation and assessment of EPAs, contrasting to previous studies conducted by O ‘Dowd et al (2019)., where primary attention was paid to internal medicine (n = 3,25%), family medicine and primary care(n = 2,17%). This finding is surprising, as the most common specialties where EPAs were developed, such as internal medicine (n = 14,36%) and psychiatry (n = 4,10%), were expected to have more follow-up studies[8], but our result shows an opposite trend. Corresponding EPAs framework and pilot project launched by the ABP and ABS might be significant drivers of this change, powered by the selected articles addressing surgery and pediatric were mostly written (n = 11, 92%) by authors from the USA, meanwhile, it is relatively easy to conduct EPAs in specialties with more emphasis on clinical skills[13].

Articles included in this review focus on four topics: The majority of the research (n = 16,55.2%) aims to verify the reliability and validity of EPAs (including various contents or tools based on EPAs, such as frameworks, mobile applications, instruments, curriculums). The second category was the description of cases or experiences regarding the implementation of EPAs (n = 11,37.9%). One interesting finding is that all the internal medicine EPAs studies and most of the pediatric EPAs studies (60%) fall into this category, probably because EPAs entries are relatively more explicit and specific, and easily implemented and evaluated in the surgical field that is highly-clinical-skill-driven. The third category contains articles aiming to gather information about factors and barriers affecting the EPAs’ implementation and evaluation(n = 6,20.6%). The last one is about research surveys on EPAs experience of related personnel (n = 5, 17.2%).

### Common ways to implement/assess EPAs in GME

#### Forms of implementation

**Figure 2.**
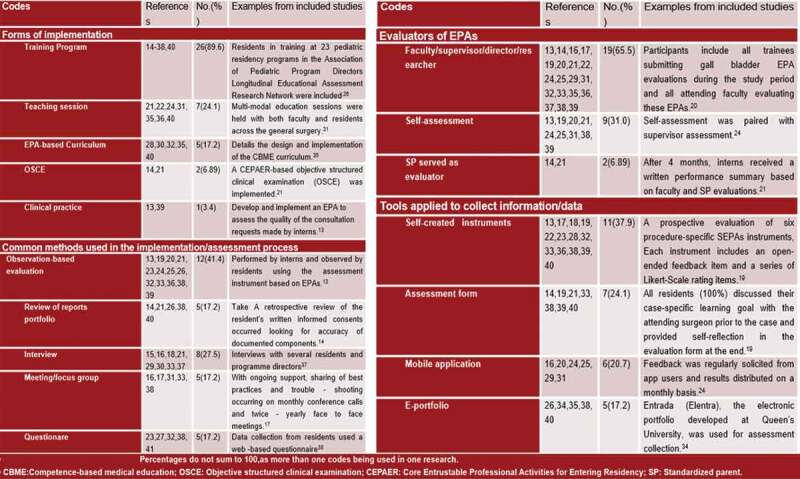
Common ways to implement or assess EPAs in GME

In the past two years, common ways of EPAs’ implementation and evaluation tend to be more diversified, shown in Figure 2. Most EPAs were being implemented in training programs(n = 26,82.7%). Five studies describe the implementation of curriculum based on EPAs(n = 5,17.2%). Compared with previous 7 year[9], the proportion of teaching sessions in programs risen from 17% to 24.1%, while the ratio of curriculums decreased from 33% to 17.2%, shown a tendency that more and more EPAs being implemented or assessed directly rather than insert into curriculums. Besides, two studies attempt to assess participants’ EPA levels by implementing objective structured clinical examination (OSCE) [14,15], which provide a new prospective for combining EPAs with existing assessment frameworks.

#### Methods used in the implementation/assessment process

There are two primary modes for evaluating the physicians’ EPAs: observation-based evaluation (n = 12,41.4%) and document review(n = 5,17.2%). Interviews are the most popular method to collect information (n = 8,27.5%). In addition, distributing questionnaires and using meetings/focus groups are two other approaches, each of which accounts of 17.2% (i.e., n = 5).

#### Evaluators of EPAs

Of all the studies included, mentors (i.e., faculty, supervisors, directors, or researchers) act as major evaluators in the assessment process (n = 19,65.5%). Among the 19 studies, nine (31%) used self-assessment methods and two (6.89%) select SPs as evaluators. The importance of adopting self-assessment should be enhanced, for it provides multi-faceted evidence to assess entrustment, and can bring physicians a greater sense of participation.

#### Tools for information/data collection

Diversified assessment tools mark a significant breakthrough in EPA practice. According to the systematic review of Emily O’Dowd et al in 2019, only two studies mentioned using assessment forms to measure physicians’ performance during 2011–2018. For now, self-created instruments and assessment forms are becoming standard tools used in EPAs studies (n = 11,37.9%; and n = 6,20.7%, respectively). Those tools usually function well and have proved to possess adequate reliability and validity [16–18].

The digitization of medical records is an irresistible trend, combined with the electronization tendency of EPAs assessment in graduate medical education. More studies choose to collect information and data using online systems. Of the research included in the scoping review, six records the assessment process using mobile applications (20.7%), five mentioned developing e-portfolio used for assessment collection (17.2%).

### Results of EPAs practice

Many studies reported the results of EPAs practice, among which 12 are longitudinal studies with vary duration from 1 month to three years. EPAs were successfully integrated into the assessment framework of training programs in GME, and feedbacks from those studies were primarily positive. Overall, EPAs were proved to be applicable mainly from four perspectives:

1. EPAs play an imperative role in promoting competency-based medical education.

EPAs were promising tools for advancing physician’s competency-based assessment and progression. It has been recognized by tremendous countries that EPAs are beneficial for transforming GME to Competency-based medical education. In the last two years, research findings further supported this opinion: EPAs showed huge potentials as a tool for assessing observational competency[19]. Additionally, as a template for devising educational settings, EPAs are effective in curriculum development and resident evaluation[20]. In the study of Stahl et al., a competency-based assessment system based on EPAs was successfully implemented, and resulted in more transparent and effective surgical training[21].

1. EPAs are valid assessment tools.

As previously stated, the use of EPAs as evaluation tools has been widely validated; In a workplace-based training context, EPAs are reliable evaluation tools applicable to both postgraduate students and faculty members[6]. According to the research, faculty members were pleased with the evaluation procedure since it reduced subjectivity and provided them with a specific framework for assessing students[22]. The comprehensive assessment of resident qualifications was highly valued by the directors, as it may offer more immediate and precise input and resolve weaknesses in the present system [23–25]. Both faculty and residents expressed favorable perspectives towards EPAs, viewing them as useful tools for providing feedback promptly following the observed patient encounter [19,26].

1. EPAs could enhance the professional capacities of participants.

The influence of EPAs in improving physicians’ ability has already been proved by many studies. A one-year longitudinal study conducted by Ju et al., found that after the implementation of EPAs, physicians showed enhanced confidence levels[27]. Kang et al. found that there is strong positive association between the resident’s appraisal of the intern’s competence to how well they use EPAs framework[28]. After the implementation of EPAs, most participants were aware of the required criteria for summative entrustment decisions. They felt more prepared[29], and showed an apparently improvement in their performance and confidence [22,25,30]. A survey on an EPA-based program found that residents generally appreciated the added value of EPAs, welcomed the opportunity of gradual advancement toward unsupervised practice and felt more motivated and focused[25].

1. EPAs are meaningful tools to ensure and improve the quality of GME.

EPAs are an excellent approach to ensure and enhance the quality of GME; it can standardize the targeted results of courses and ensure that no resident was left behind[31]. In a study, 89% of interns claimed that they would make a change according to EPAs feedback in their next consultation request[28]. Similar evidence is provided by two other studies: in the first one, participants agreed that EPAs met the defined criteria and were considered important for graduates to be able to demonstrate the expected behaviors[32]; in the second one, the majority of the physicians agreed that the assessment based on EPAs was meaningful, and immediate feedback was beneficial[15]. Another 3-year longitudinal study claims that EPAs serve as an effective channel for communicating expectations to resident participants[20].

Nonetheless, certain issues are raised in those studies, the most prominent of which being the appropriateness and fairness of EPAs. Other research have indicated that physicians had a poor actual performance rate. In the research of Holzhausen et al., only 3 out of 13 EPAs procedures had been achieved by most of the graduates under indirect supervision. None of the 5 advanced EPAs had been performed by more than 75% of the participants[33].

Another significant issue is that several physicians raised concerns about the fairness of EPAs. When EPAs are deployed as measurements of performance and eligibility for promotion or graduation, the fairness of the system needs to be heavily scrutinized[34]. In one study, surgery resident self-evaluations agreed more strongly with surgery faculty assessments than with emergency medicine faculty assessments, highlighting the importance of choosing suitable evaluators[35]. In the survey of Baer, most physicians were anxious about being evaluated by someone they do not know or who may have just seen a portion of their patient engagement[19]. In addition, the issue of comprehensiveness of EPAs as an assessment tool is of concern. To be concrete, Solymos et al. examined faculty feedback reports and discovered that participants seemed to favor clinical parts of an EPA over non-technical areas[13]. Moreover, several components of preoperative or postoperative care are not observable, resulting in intraoperative assessment ruling the EPA evaluation[36].

### Challenges of EPAs practice

Although EPAs are utilized satisfactorily at many institutes, the practice of EPAs is still fraught with difficulties. First, the acceptability of various medical systems to EPAs, as well as the costs of EPAs, are always enormous, and both may be prohibitive in terms of time and economic burden. There is a need for increased faculty development on how to use the tools during the process [24,34,37]. For certain developing countries, such as India, there was a severe scarcity of manpower and resources, and management had to pay close attention to actual conditions[31]. On the other hand, the adoption of EPAs could be hampered by legal problems in countries such as the Netherlands[25]; this phenomenon also exists in China: residents must participate in at least one year of standardized training in addition to completing the national medical exam and receiving a Medical Practitioner’s Qualification Certificate; during this time, they are not legally permitted to diagnose patients independently, regardless of how well they have done in practice.

The second challenge is the acknowledged level of EPA evaluation results. According to the studies, EPAs need to be modified to account for changes in healthcare systems and training programs. Instead of being extrapolated from one environment to another, EPAs must be properly built for their intended context[6]. However, this raises the question of whether EPAs generated by one institute will be recognized by another. Smit et al., discovered that recurrent doubt occurred to recognize the EPAs received in another clinical unit, department, or institution. This study also discovered trust concerns between instructors and residents[25]. Another study discovered that, while nominal entrustment levels improved with each year of training, the actual improvement remained undetermined; the EPAs tasks were in usual deliberately simplified, since the involvement of real patients could be highly sensitive and therefore only those of ‘easier’ cases were adopted in the assessment[17]. Given the significance of patient safety in medical education and the trust connection between physicians and patients, some academics propose that the role of the patient in entrustment decisions be investigated as well[6].

Adaptation of both physicians and instructors to EPAs is also a significant challenge for the two parties. One of the effects of EPAs is that they create uncertainty by affecting the development of competence and the frequency with which activities are performed, requiring more time to utilize and practice at the first stage. As a result, the process per se produces a great deal of stress among physicians and instructors [32,38]. It is also likely that residents may prioritize the EPAs over other important tasks and education[36]. The core of EPAs is learner-centered education, requiring them to possess fine self-management ability, which was proved not easy in many studies [31,36]. Finally, concerns that EPAs being highly subjective and potentially harmful to underrepresented groups (e.g., minority) was claimed[34].

## Discussion

The use of EPAs in medical education has gradually advanced from the construction of frameworks to the follow-up of their implementation and evaluation in clinical practice. This study is the first scoping review specifically on the effectiveness of EPAs’ implementation and evaluation in GME. We synthesized 29 recent research studies (published between 2019.01 and 2020.12) to provide an overview and analysis of the common approaches, implementation effectiveness, difficulties and challenges faced in EPAs implementation and evaluation. This review will be useful for faculty involved in EPAs practice, physicians, and for institutions and researchers proposing to conduct EPAs.

The ways of implementing EPAs are gradually diversifying. There is no standardized process for implementing EPAs from existing research, but this review provides an important overview of the EPAs implementation process that may provide a methodological reference for future EPAs implementation. Based on the results of the existing studies, the following components are common in the implementation of EPAs: Teaching session, Training program, Faculty evaluation (Observation-based evaluation or/and Review of reports), and Self-assessment. However, of all the studies included in this review, only two included all these processes [15,24].Although some papers have confirmed the importance of conducting a teaching session for participants to accurately understand and successfully implement EPAs [13,15,21,22,24,35], only seven of the selected literatures offered this session prior to implementing EPAs. A few studies found that physicians performed poorly on EPAs and that teachers or physicians were not familiar with the framework and scope of EPAs [14,19,26], probably due to the absence of an introductory session on EPAs like a teaching session. However, it has also been suggested that the reason for not providing training to attending surgeons, residents and observers is to reduce potential cognitive biases[16],an assertion that has yet to be tested. Therefore, future research should focus on the impact of differences in EPAs implementation sessions on the effectiveness of EPAs, and then to develop standardized guidelines for EPAs implementation. The information on EPAs implementation methods compiled in this review may serve as a reference for the development of standardized EPAs implementation guidelines.

An important purpose of EPAs is to facilitate the shift to a learner-oriented model, while half (9 of 19) of the selected EPA studies involved both instructor-assessment and self-assessment, the results were still primarily presented in the form of instructor-assessment. There is research evidence that physicians feel more confident and motivated to focus on specific tasks and feel more engaged in their own learning process, because of participating in EPAs. [17,25] However, there are still no empirical studies on this issue. There is a need to design more studies to explore the interaction between the degree of physicians’ dominance in the evaluation and their competence development, and to provide evidence to support the optimization of the effectiveness of the implementation of EPAs in the future

As shown in the *Results of EPAs practice* section, several results on the implementation and evaluation of EPAs indicate the positive impact of this assessment approach on participants[28],in terms of effectively guiding the learning assessment process [17,37],or demonstrating superiority over other competency frameworks[23].

However, an over-reliance on the evaluation function of EPAs may result in the neglect or exclusion of other activities not covered by EPAs. To circumvent this problem, a comprehensive and rigorous program of EPAs must be developed, and this requirement for comprehensive and refined EPAs conflicts to some extent with the original intention of utilize EPAs to reduce the burden on faculty in CBME assessment[17]. Developing an EPAs program that balances validity and workflow-simplification is not easy for designers and participants. Strengthening the role of EPAs as a supplementary learning tool and weakening influence of their assessment and evaluation results on physicians’ careers may become an important way to prevent this drawback, which is to be verified by future studies.

Although EPAs have become more imperative in the medical education globally, with one hundred relevant publications in 2019 alone, this study argues that the implementation and popularization of EPAs on a larger scale is still a long way off. First, from the available studies, the cost of constructing and implementing EPAs is huge, the planning period is long, and it requires the integration of educational experts, medical experts, faculty, residents, and others. Second, like CBME, the corresponding result of refining the standards (to a more detailed level) is weakening the applicability of EPAs. Furthermore, as a formative assessment tool, EPAs can track and compare participants’ competency levels at different stages, requiring a certain degree of fixity in the EPAs assessment system; meanwhile, it is worth further research on how to achieve timely updates to meet the needs of competency growth at different stages.

## Limitations

Like other studies of this kind, this review contains limitations. Firstly, due to resource limitations, only papers published in English were included; secondly, the review focused only on EPAs for GME physicians. This decision was made mainly because of the more urgent need for reform in the current Chinese residency training segment compared to other phases. Focusing on the effective coherence between institutional education, postgraduate education, and continuing education, and exploring evaluation methods that track medical students’ careers and aid lifelong learning are urgent breakthroughs in subsequent studies.

## Conclusions

This review offers suggestions for future EPAs-related research. First, the value of EPAs implementation and evaluation must be examined from a benefits perspective; the economic and time costs of a complete EPAs construction, implementation, and evaluation process are huge, increasing the workload of the participants, resulting in the need for optimizing the process in the future; Second, there are few studies that present in detail the feedback of stakeholders and the process of further designing specific improvement programs based on the corresponding feedback, i.e., it is still unclear how to optimize for poor feedback; Third, the evaluation of the EPAs framework is mainly from subjective evaluations such as interviews and questionnaires, and more quantitative studies on the effectiveness of the EPAs evaluation system are needed in the future, including a comparison of the results of EPAs and existing evaluation tools. Finally, there is no research on the migration of a more mature EPAs framework to different environments which is critical for the popularization and promotion of EPAs. In addition, existing studies are mainly concentrated in Europe and the USA, and there is still a lack of relevant studies in Asian countries.

## Appendix

**Screenshot of Web of Science Results of Related Keywords**
